# A Functionally Relevant Tool for the Body following Spinal Cord Injury

**DOI:** 10.1371/journal.pone.0058312

**Published:** 2013-03-06

**Authors:** Mariella Pazzaglia, Giulia Galli, Giorgio Scivoletto, Marco Molinari

**Affiliations:** 1 Department of Psychology, Sapienza University of Rome, Rome, Italy; 2 IRCCS, Fondazione Santa Lucia, Rome, Italy; Royal Holloway, University of London, United Kingdom

## Abstract

A tool such as a prosthetic device that extends or restores movement may become part of the identity of the person to whom it belongs. For example, some individuals with spinal cord injury (SCI) adapt their body and action representation to incorporate their wheelchairs. However, it remains unclear whether the bodily assimilation of a relevant external tool develops as a consequence of altered sensory and motor inputs from the body or of prolonged confinement sitting or lying in the wheelchair. To explore such relationships, we used a principal component analysis (PCA) on collected structured reports detailing introspective experiences of wheelchair use in 55 wheelchair-bound individuals with SCI. Among all patients, the regular use of a wheelchair induced the perception that the body’s edges are not fixed, but are instead plastic and flexible to include the wheelchair. The PCA revealed the presence of three major components. In particular, the functional aspect of the sense of embodiment concerning the wheelchair appeared to be modulated by disconnected body segments. Neither an effect of time since injury nor an effect of exposure to/experience of was detected. Patients with lesions in the lower spinal cord and with loss of movement and sensation in the legs but who retained upper body movement showed a higher degree of functional embodiment than those with lesions in the upper spinal cord and impairment in the entire body. In essence, the tool did not become an extension of the immobile limbs; rather, it became an actual tangible substitution of the functionality of the affected body part. These findings suggest that the brain can incorporate relevant artificial tools into the body schema via the natural process of continuously updating bodily signals. The ability to embody new essential objects extends the potentiality of physically impaired persons and can be used for their rehabilitation.

## Introduction

Many physiological and psychophysical studies suggest a highly complex relationship between the body and relevant extracorporeal objects [1]–[3]. In humans, tool use induces plasticity after both short- and long-term learning and practice [4], and therefore, perceptual, motor, and cognitive capacities [5] are reformed based on the mode of use [6]. Specifically, if a tool extends the able body’s movement potential, the object becomes part of the body (a process known as “embodiment”) [4], distorts the perceived body dimension [7], and alters the sensorimotor state that guides actions [2], [8].

These bodily changes may result in either a conscious, visual representation of the manner in which the body is perceived, known as “body image” [7] or, on the other hand, potentially update unconscious sensorimotor representations to enable motor control, as referred to as “body schema” [9]. Behavioral studies have specified that, although seeing and touching a tool affirms its embodiment, movement may not be necessary (e.g., a rubber hand can be embodied without moving it) [10]. However, the ability to control movement enhances the feeling of embodiment [11], whereas the inability to control movement prevents it [12]. When a prosthetic device is used for action and constrains the injured physical body to a new position, the appropriate extracorporeal tool may be assimilated as a corporeal structure [8], [10], influencing the body schema and body image [7]. For example, patients paralyzed because of spinal cord injury (SCI) may lose movement and sensation permanently, thus becoming dependent on a wheelchair for mobility and changing their body posture drastically. Influential theoretical models [13], [14] and empirical studies [7], [15]–[20] have suggested that, in these cases, the body schema and body image are rearranged to incorporate the wheelchair. The experience of wheelchair embodiment has not been evaluated using quantitative measurements but, rather, through systematic descriptions of patient experiences. No definitive conclusions have been drawn from these studies and reports concerning wheelchair embodiment are controversial. Although some patients with SCI experience the wheelchair as a corporeal structure, others regard it as an artificial device. For example, a male patient who had a complete lesion at the fifth cervical vertebra was able to flex his elbow but was completely paralyzed from the chest down and had no hand movement, reported, “[The wheelchair] is not a part of me. It might need to fit me like a pair of trousers; it might need to be there when I want it to do what I want to do, but it is not a part of me” [21]. Conversely, a 27-year-old male patient with a thoracic lesion, complete paralysis of the lower half of the body, and spared hand movement and sensation said: “It is a part of me… I forget it” [13]. In these two cases, patients with upper and lower SCI reported differences in the corporeal experiences with the wheelchair.

Generally, injury to the upper level of the spinal cord results in greater deficits than injury to the lower level. Cervical spine lesions induce legs and trunk paralysis as well as a variable degree of sensory loss and partial paralysis of the upper limbs. In contrast, lesions of the thoracic and lumbar spine cause paralysis of only the lower limbs. Given the preservation of cognitive functions in SCI patients, the mobility-impaired wheelchair-bound patients offer a unique opportunity to characterize the inherently plastic nature of the body schema and body image as a result of tool use. In particular, sensorimotor deprivation and the specific use of a wheelchair may modulate the development of the corporeal awareness of a tool. Therefore, bodily representation can change because of temporary (regional anesthesia) [22], [23] or permanent (amputation and peripheral nerve lesions) [24], [25] modification of sensorimotor signals. It is logical to expect that the proportion of the body that is “isolated” from the brain may have an impact on the embodiment of a tool. Along the same lines, the systematic adaptation to an assistive device requires a change in the body’s center of mass [7]. Thus, long-lasting distortions of the body morphology may reflect a new state of the body image, leading to a coherent modification of corporeal awareness.

In this study, we collected structured reports on the introspective experiences of regular wheelchair use in patients with SCI. Our aim was to determine, after SCI at different levels, how the degree of spared sensorimotor function or the prolonged confinement by sitting in a wheelchair modulates the introspective experiences of instantiation of a wheelchair as captured by a standard principal component analysis (PCA).

## Methods

### Participants

Fifty-five wheelchair-bound patients were recruited from the Santa Lucia Hospital in Rome, where they were undergoing treatment in the Spinal Cord Rehabilitation Unit. All patients navigated autonomously in their wheelchair, using their arms for control. Three patients with complete cervical injuries (patients 1, 2, and 17) operated an electronic wheelchair. The remaining 52 patients propelled a wheelchair manually. The patients utilized their wheelchairs for approximately 13 h/day.

### Assessment of Individuals with SCI

**Figure 1:**
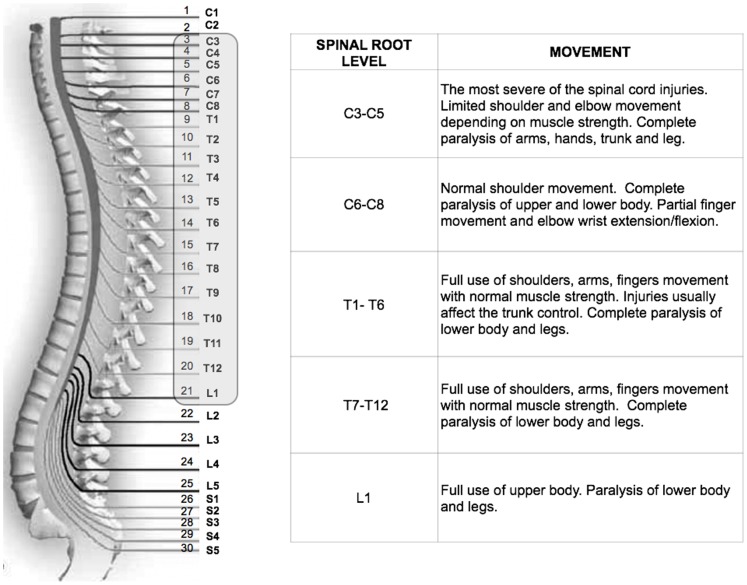
Relationship between the nerve level of the SCI and movement. Among all patients with SCI recruited for this study, the level of lesions ranged from C3 to L1, as highlighted in the figure.

The SCI lesions ranged from C3 to L1, as shown in Figure 1. Lesions were at 92.2±84.6 months post-SCI (range: 6.2 to 340.6 months), which is within the chronic injury phase. None of the patients had experienced head or brain lesions associated with their SCI, as documented by magnetic resonance imaging (MRI). A neurologist (G.S.) examined each patient after admission to the study. The international standards of the American Spinal Injury Association (ASIA) for the classification of SCI were used to document the sensory and motor impairments following SCI [26]. The third version of the Spinal Cord Independence Measure (SCIM III) was used to quantify the functional status of each patient [27], [28]. For the purposes of this study, the Self-care and Mobility subscales were used. The Self-care subscale consists of six items with scores ranging from 0 to 20. The Management and Mobility subscale consists of nine items with scores ranging from 0 to 40. Grades of 0 to 8 (requiring higher ability) are assigned for each item according to increasing difficulty. The neurological and clinical data are presented in Table S1 in File S1.

### Ethical Statement

Written informed consent was obtained from all participants. The study protocol was approved by the local ethics committee (IRCCS Ethics Committee at Fondazione Santa Lucia, Rome; Protocol CE/PROG.309-16) in accordance with the ethical standards of the Declaration of Helsinki.

### The Relationship between Injury Location and Functional Disorder

After spinal cord injury, the body parts located below the point of the lesion are insentient and unmoving. The various degrees of sensory and motor impairment are expressed mainly by neurological status, which is determined by the level and severity of the damage to the spinal cord.

Accordingly, the spinal cord vertebrae were numbered from the top, beginning at the neck and extending downward through the back. Lower numbers indicate upper cervical vertebrae (Figure 1). The lesion level was converted to a numerical value of the corresponding numbered vertebral column level. The neurological status determines the number of body segments that are “isolated” from the brain and the functional activity or potential ability. Indeed, significant positive correlations were observed between the lesion level and the Self-care subscale scores (Spearman *r*_(53)_ = 0.68, *t* = 6.71, *p*<0.0001) and Management and Mobility subscale scores (Spearman *r*_(53)_ = 0.59, *t* = 5.30, *p*<0.0003), indicating a direct relationship between the level at which the spinal cord lesion occurred and the degree of body functional capacity. Although the patients with upper spinal cord lesions included in our study retained partial upper-body function, injury in the upward-extending region of the spinal cord reflects a higher degree of sensory loss and partial paralysis, which consequently modulates the overall ability to act in a wheelchair.

### Questionnaire

Using a rating scale ranging from 0 (“completely disagree”) to 7 (“completely agree”), participants evaluated questions designed to capture the implicit and explicit tool and body experiences. The questions, including previously adapted hypothesized constructs with prosthetic devices [24], were selected on the basis of a previous analysis of the transcripts from informal interviews with 13 patients with SCI who reported wheelchair-related feelings (unpublished). Six of the questions concerned the implicit (items BI_1_–BI_6_), and five questions explored the explicit (items BE_1_–BE_5_) bodily experiences of wheelchair use. The following questions translated from Italian were investigated:

[BI_1_] Diet: Do you follow a controlled diet to prevent changes to your body shape and to avoid problems with the wheelchair?

[BI_2_] Maintenance: Do you think of ways to prevent problems with the wheelchair? That is, do you pay particular attention to its maintenance?

[BI_3_] Defense: Do you protect your wheelchair from dangerous situations?

[BI_4_] Awareness: Did you experience any change in your attention and/or awareness while being in a wheelchair (after 1, 3, and 6 months)?

[BI_5_] Tool: Do you perceive the wheelchair as an external tool?

[BI_6_] Affect: Do you feel emotionally attached to your wheelchair?

[BE_1_] Entire body: Do you perceive the wheelchair as part of your entire body?

[BE_2_] Lower limbs: Do you perceive the wheelchair only as part of your lower limbs?

[BE_3_] Substitution: Do you perceive the wheelchair as a “substitute” for your body?

[BE_4_] Extension: Do you perceive the wheelchair as an “extension” of your body?

[BE_5_] Action: Do you perceive the wheelchair as a form of compensation for your actions?

Having defined implicit and explicit body experiences with the wheelchair, we next specifically targeted the presence or absence of corporeal awareness of the wheelchair by using the following two questions:

[BI] Image: Close your eyes and imagine yourself [pause for 3 s]. Do you see the wheelchair?

[BE] Frame: When thinking about your body frame, do you feel that the wheelchair is an internal part of your body?

### Statistical Analyses

Wheelchair embodiment among SCI patients was determined based on 11 different questionnaire statements. A PCA with an orthogonal varimax rotation was conducted to reduce the dimensionality of the data by computing new variables called principal components, which were obtained as linear combinations of the original items.

Certain indicators are traditionally used to draw conclusions regarding the appropriateness of a PCA. The strength of the linear relationship between items has been represented by a correlation coefficient greater than 0.3 [29]. Although the ratio of patients to items was 5∶1, as recommended [30]–[34], we also used the Kayser–Meyer–Olkin (KMO) measure to test the adequacy of the sample and the Bartlett test of sphericity to verify the extent of correlation allowable between items. Scree plots and eigenvalues greater than 1 were used to determine the appropriate number of components. Only items that loaded strongly (above 0.5) were considered, in accordance with the standard PCA approach [35] and psychometric evaluations of embodiment [36].

Briefly, the different steps involved in a PCA include the calculation of the correlation matrix, the extraction of the initial principal components, the application of the varimax rotation, the calculation of factor scores assigned to components, and the generation of factor loadings weighted for each component extracted. On each principal component axis, we also computed a single score to which all normalized measurements contributed for each patient.

We then analyzed the factor scores addressed in the PCA using a multiple regression analysis to explore the embodiment facets that are related to the clinical data (i.e., lesion level or exposure to/experience with the wheelchair).

## Results

We will first report a brief summary of the results obtained for each question regarding the presence or absence of a corporeal attribution of the wheelchair. Among the 55 participants, 67% experienced the feeling that the wheelchair was integrated with their body [BE question], and 72% viewed the wheelchair in their corporeal image [question BI]. The percentage of responses indicating the presence of the wheelchair within the boundaries of the physical body in answer to at least one of the two questions described above (BE and BI) was higher than the percentage of responses indicating its absence (binomial test, *p*<0.04). These percentages of wheelchair assimilation served as a “phenomenon check.” We then analyzed the questionnaire statements using the PCA.

**Figure 2:**
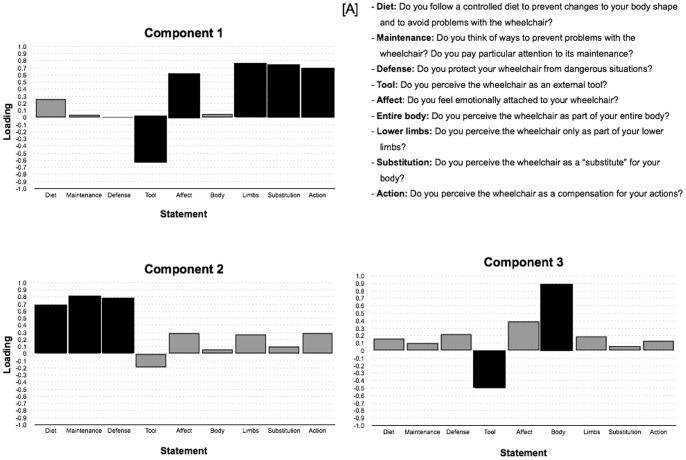
Loadings of the statements on the three principal components extracted. The labels on the x-axis refer to the statements shown in panel A. Black bars indicate the statements with the highest loadings (≥0.5) for each component. **A.** Statements used to assess wheelchair embodiment.

With the exception of two items (BE_4_ and BI_4_) all other correlations were significant at the 5% level and entered in the PCA analysis. The KMO test yielded values (KMO = 0.68) above the acceptable limit of 0.5 [37]. Moreover, Bartlett’s test of sphericity indicated that correlations between items were large enough for the PCA χ^2^_(36) = _119; *p*<.0001). Analyses of eigenvalues and scree plot converged in the extraction of three components that, together, accounted for 66.3% of the variance. Figure 2 shows the statement scores for each of the three principal components. The means and standard deviations and communalities for the statement scores are given in Table S2 in File S1.

Principal component 1 accounted for substantially more variance than the other two components (variance = 31.01%; eigenvalue = 2.8) and included items BI_5_, BI_6_, BE_2_, BE_3_, and BE_5_, which exhibited the highest loadings (magnitude of 0.5 or more). A major difference was observed in the positive loadings for “action,” “lower limb,” “substitution,” and “affect” and in the negative loadings for “tool,” suggesting that two separate processes load on the first component. The wheelchair appeared to be processed as if it were a part of the patients’ limbs as opposed to a tool that reflects a more substitutive process linked to actions. This is consistent with the concept of functional embodiment. Although the positive loading for “affect” may seem to be a functionally less relevant point, this may partly explain the association between the level of wheelchair use and satisfaction of patients with their tool.

Principal component 2 (variance = 20.6%; eigenvalue = 1.85) included items BI_1_, BI_2_, and BI_3_, which captured the burden of assistive tool care. The new corporeal state leads to the management of body weight, and the safety, risks, and dangers that redefine the person in terms of “body plus wheelchair” gain more focus.

Principal component 3 (variance = 14.4%; eigenvalue = 1.3) included items BI_5_ and BE_1._ These statements are related to the perception of the wheelchair as a body part (positive loading) as opposed to a tool (negative loading). Item BI_6_ loaded moderately (0.40) on the component 3 providing convergent evidence that a lower emotional attachment to the wheelchair selectively influences the sense of embodiment of the tool.

We also computed the 55 individual component scores, using a single composite measure created for each patient on each orthogonal dimension. A factor score represents a participant’s standard score on each specific component.

To identify the potential predictors of the three components, we investigated the relationship between the factor scores of each component and the clinical data: the lesion level, the time since the lesion, and exposure to (daily hours) and experience (time since use) with the wheelchair. The multiple linear regression analysis revealed that a lower lesion level predicted a larger value for principal component 1 (β = 0.48, F_(4,50)_ = 4.4, *p* = 0.004). Lesion level was not a significant predictor of components 2 (β = 0.19, F_(4,50)_ = 1.01, *p* = 0.40) or 3 (β = −0.06, F_(4,50)_ = 0.95, *p* = 0.44). Among all SCI patients, neither the time since injury nor exposure to/experience with the wheelchair predicted the individual component scores for each of the three principal components (β = n.s. for all). The model indicated that having a lower lesion enhanced the positive factor (limb, action, and substitution) linked to the functional aspect of the embodiment. However, this relation was reversed for the tool factor, which exhibited a negative score. This linear relationship suggests that the feeling of functional embodiment regarding the wheelchair should be substantially enhanced in relation to active body segments.

The sensory-motor control of a wheelchair imposes considerable demand on the upper extremities. In particular, a precise balance occurs between the full use of the upper limbs and the strength or endurance of the trunk muscles to guarantee stability [38].

All SCI patients in the study had complete paralysis of the legs but had various degrees of upper body impairment, such as hand/arm and trunk deficits. To more fully determine the demands on the specific upper body segment, we categorized patients with complete (grade A) injuries into tetraplegia (T: C_3_–C_7_), high paraplegia (P_H_: T_2_–T_7_), and low paraplegia (P_L_: T_8_–L_1_) groups. The first group included patients with extensive deficits in the entire body (in the arms as well as the trunk). The second group included patients with full use of hands/arms but limited strength, balance, and use of the trunk. The last group comprised patients who had more full use of the upper body (arms and trunk).

A mixed-model ANOVA with significant items in principal component 1 (BI_5_, BI_6_, BE_2_, BE_3_, and BE_5_) × group (high-paraplegia, low-paraplegia and tetraplegia) revealed a significant effect for the items type (F_(4,164) = _6.7; *p*<0.001) which was explained by the higher ratings for BE_5_ (*p*<0.01 for all) compared with the other items (BI_5_, BI_6_, BE_2_, BE_3_). No significant differences were observed between groups (F_(2,41)_ = 2.14; *p* = .13).

However, we did observe a significant group × item interaction (F_(8,164) = _2.8; *p*<0.006). Fisher’s post-hoc test revealed that individuals with low and high paraplegia had a significantly higher rating when perceiving their wheelchair as part of their lower limbs (P_L_ = 4.41 and P_H_ = 4.11) than those with tetraplegia (T = 2; *p*<0.04 for all). Interestingly, patients with tetraplegia tended to regard the wheelchair more as an external device (T = 4.7) compared with individuals with low paraplegia (P_L_ = 2.6; *p*<0.01) but not compared with those with high paraplegia (P_H_ = 3.2; *p*>0.11). Patients with low paraplegia (P_L_ = 5.94; *p*<0.04) tended to regard the wheelchair more as a compensation for their actions compared with individuals with tetraplegia (T = 4.3) but not with those with high paraplegia (P_H_ = 5.5; *p*>0.68); the two latter groups did not differ from one another (*p*>0.21). Upper extremity interaction with the wheelchair enhances the feeling of embodiment. The trunk, which functions to maintain posture and partially govern wheelchair movement, appears to modulate the flexibility in the integration of the tool.

**Figure 3:**
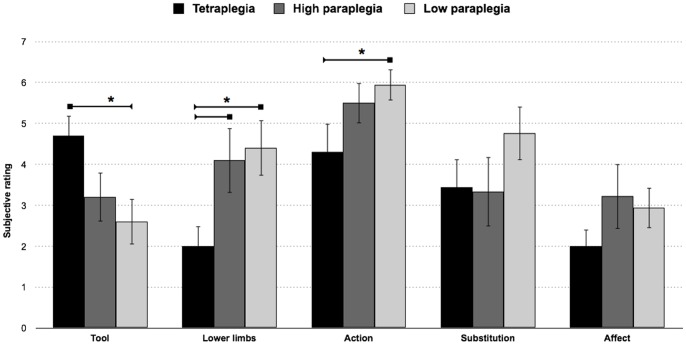
Functional aspect of the sense of embodiment concerning the wheelchair. The mean subjective ratings for the statements with the highest loadings in Component 1 in the three subject groups with complete injuries (tetraplegia, high paraplegia and lower paraplegia). The error bars indicate the standard error of the mean (SEM). The asterisks (*) indicate significant results from the post hoc comparisons (p<.05).

We observed no relevant effects of the other statements (affect and substitution), which indicates that the three groups considered these aspects of the embodiment experience with a wheelchair in a similar way, despite their different lesions and body capacities (see Figure 3).

## Discussion

Most patients with SCI perceive that their legs are still their own [39], despite their inability to use or feel them [21]. Although the patients do not confuse their body parts with their wheelchair [40], some do consider themselves to be “individuals with a wheelchair,” whereas others regard themselves as “enwheeled individuals” [13], [21]. This identity or “wholeness” discrepancy prompted us to investigate whether the somatomotor deafferentation/deefferentation of disconnected body segments and the exposure to/experience with the tool, affects corporeal awareness of the wheelchair. To explore the presence of any such relationships, we developed a novel questionnaire regarding wheelchair-related feelings.

Among all participants included in the present study, a significant number experienced the wheelchair as being internal to the corporeal boundary, suggesting a revision in their body image. The perception of the body’s edges does not appear to be fixed; rather, the body is plastic and flexible to assimilate the tool. As captured by component 1, the corporeal awareness of the tool emerges not merely as an extension of the body but as a substitute for (and part of) the functional self. This assistive device offers the possibility, at least in principle, to partially “repair” the motor functionality of the damaged body part [24], [41] and appears conceived not as an object to move but as a mediator of the limbs’ action. This reorganization of body model is consistent with the positive inclusion of the wheelchair to accommodate physical impairment and restore mobility [13]–[15], [18], [42], [43]. The perceived bodily experience is that of being functionally whole, and the system reorganizes itself to achieve its original balance, which enables the immobile user to act in the world. Amputees who use prostheses, which are less efficient and less safe than a wheelchair, report that the object became “part of them,” and they feel as though they have a normal, complete body [24], [44]. Importantly, the emotional and physical acceptance of and adaptation to the wheelchair occurs over a period of years [45], [46]. It also appears to affect the new corporal state whereby the tool feels like “part of” the user. The wheelchair requires the regulation of weight and a great deal of effort and control (maintenance and defense) to achieve reliable usage. The burden of assistive tool care appears to be indirectly processed considering the safety, risks, and danger to the body.

Given the prolonged history of immobility, the bodily attribution may refer to confinement in the wheelchair. However, no effects of either exposure to or experience with the wheelchair on the embodiment of this tool were observed. In contrast, we found that chronic sensorimotor loss specifically predicted the individual’s corporeal awareness of his/her wheelchair.

A more unconscious body model, the body schema, which enables motor control and reflects the proprioceptive, tactile inputs of how the body is “felt”, may regulate the functional aspects of embodiment considerably. Indeed, the compensatory flexibility of wheelchair embodiment observed in patients with SCI is linearly linked to their ability to feel and move the superior extremities, the trunk and arms in particular.

Accordingly, it has been suggested that the embodiment of an object is modulated by tactile interaction [47], and objects that have been in contact with the body [48], [49] and are actively used [6] become part of the bodily representation [4], [6]. The online information regarding movement in a wheelchair is a prerequisite for the capacity to feel that the event is generated by one’s own body and one has control over it. The different modes of wheelchair use (from placing the hands on the wheels to steering a knob in more severe cases) may reflect a different attribution or evaluation of being the author of the movement, affecting how the retrospective sense of agency is perceived. This prediction might be tested in future research by comparing patients with SCI who operate their wheelchair manually with those who operate it electronically.

In the case of an upper spinal cord lesion, much more than in the case of a lower spinal cord lesion, there is a more pronounced reduction of strength and functionality in the entire body as well as an overall lack of feeling of touch. Such impairment interferes with the feeling of the wheelchair in direct contact with the body and with other objects and mainly with the regular status that updates the enwheeled body in motion. This failure to “capture” the somatic, proprioceptive, and motor information continuously being exchanged with the “body plus wheelchair” hinders the processes that are essential for creating an abstract reference of the body frame [50], [51], leading to the feeling of the wheelchair as a corporeal assimilation. This concept is in line with the particular aberration of corporeal detachment and distance observed in patients with higher SCI [39] as well as in individuals with locked-in syndrome [40].

Neuroanatomically, such distorted sensorimotor input presumably induces adaptive or maladaptive cortical reorganization [52]. After SCI, the loss of afferent/efferent information related to the body parts located below the lesion leads to the structural and functional reorganization of the cortex, particularly in somatomotor areas [53]–[55], and affects complex intracortical connections [52], [54], [56]. Decreased frontal and frontoparietal cortical connectivity by the alteration of ascending and descending neural information flow is most pronounced in individuals with upper SCI [57]. Conversely, in patients with lower SCI, an expansion of the primary somatosensory and motor-cortex hand area into the output-deprived primary-cortex leg area was observed [55], [58], which translates into a functional gain for the internal sensorimotor body representation [52].

Moreover, atypical connectivity plays a prominent role in neuronal activity within the parietal cortex, which is the dominant structure for bodily representation [59]. Indeed, recent clinical and neuroimaging data suggest that temporoparietal junction (TPJ) activity reflects the multisensory integration of bodily instantiation [60] as well as feelings of spatial unity related to the body [61]. Moreover, the TPJ is thought to rely on the combination of tactile and proprioceptive information in a coordinated reference frame [62], [63]. Dysfunction in this area may lead to a modified body experience, which is felt to be spatially disconnected [64]. Nevertheless, without further data, we cannot discern whether the modulation of embodiment results from effects on brain networks, the periphery, or both. This should be investigated in future studies, for example, by investigating changes of the BOLD signal in brain areas of bodily representation in both injured and healthy subjects using paradigms eliciting self-referential activity during the observation of an avatar engaged in dynamic actions with tools.

One potential study limitation was the use of introspective data and PCA, which, although an elegant and powerful tool [36], needs to include empirical measures. Therefore, we aimed to establish the effect of sensorimotor loss, and the specific use of this tool, on wheelchair embodiment. It is important to note that previous SCI studies have already demonstrated the physical adjustment to, [7], [15], [16], [18] and brain representation of [17], the “body plus wheelchair” being perceived as one. We also capitalized on the fact that 12 of the patients recruited for this study were tested in a separate experiment that indicated patients with SCI embody functionally relevant wheelchair action sounds (unpublished data, presented in Galli et al., Concepts, Actions, and Objects; Functional and Neural Prospective Meeting Abstract, 2012).

Altogether, our data suggest that the subjective experience of the embodiment of an external tool in patients with SCI is a complex, multifarious process that requires the following: a feeling of ownership over the tool (including a long-lasting coherent and accurate representation); online multisensory integration, referenced on the state of the body (including the effective regulation of sensorimotor information flow); and, finally the self-attributed control of the physical body and its movement.

The phenomenal reports from SCI individuals cannot be generalized to all occurrences of corporeal awareness of a tool but offer an initial step towards the determination of clearly dissociable subcomponents of prosthetic device embodiment. Indeed, the objective and quantitative evaluation of changes in patients with spinal cord lesions help identify the cause that may preclude the experience of self-attribution and embodiment of a tool. United harmony between the body and the tool may be key for the embodied experience of success or rejection of an assistive device. Embodying a wheelchair may enhance the efficiency and safety of movement, thereby reducing bodily effort and the damage produced by its use. This ease of use may lead to greater autonomy and self-organization, thus allowing patients to benefit from the opportunities offered by the environment in which they move.

## Supporting Information

File S1 **Table S1. Clinical and demographic characteristics of patients with SCI.** The neurological levels of the lesion and injury, as determined using the American Impairment Scale (AIS), are indicated. Spinal Cord Independence Measure (SCIM) scores were not available for patients No. 12 and 25. **Table S2. Scores and communalities on questionnaire statements.** Mean, standard deviations, and communalities for the three components in each of the statements.
(PDF)
