# Supplementary material for: A Functionally Relevant Tool for the Body following Spinal Cord Injury
Source: PLoS One. 2013 Mar 6;8(3):e58312. doi: 10.1371/journal.pone.0058312 (PMC3590178; doi:10.1371/journal.pone.0058312)
Supplement: File S1 — Table S1. Clinical and demographic characteristics of patients with SCI. The neurological levels of the lesion and injury, as determined using the American Impairment Scale (AIS), are indicated. Spinal Cord Independence Measure (SCIM) scores were not available for patients No. 12 and 25. Table S2. Scores and communalities on questionnaire statements. Mean, standard deviations, and communalities for the three components in each of the statements. (PDF) [file pone.0058312.s001.pdf]

TABLE 1

DEMOGRAPHIC AND CLINICAL DATA OF THE PATIENTS

Case	Age	Gender	Time since Injury (days)	Time since wheelchair utilization	Daily wheelchair use (hours)	Lesion Level	AIS grade	Etiology	SCIM Self-Care (0-20)	SCIM Mobility (0-40)
P1	26	F	3350	3260	10	C3	A	Traumatic	0	3
P2	30	M	2550	2450	10	C4	A	Traumatic	0	3
P3	40	M	730	715	15	C4	D	Traumatic	20	20
P4	24	M	1654	1530	11	C4	B	Traumatic	1	6
P5	30	M	4015	3960	15	C5	A	Traumatic	18	14
P6	35	M	258	195	10	C5	A	Traumatic	4	6
P7	29	F	8395	8310	10	C5	C	Traumatic	-	-
P8	29	M	540	450	12	C5	A	Traumatic	13	13
P9	40	M	980	920	10	C5	A	Traumatic	4	5
P10	25	M	975	900	15	C5	A	Traumatic	12	11
P11	22	M	420	350	11	C5	A	Traumatic	11	11
P12	31	M	3650	3600	14	C6	A	Traumatic	14	16
P13	40	M	6570	6500	15	C6	A	Traumatic	18	16
P14	54	F	8230	8160	12	C6	A	Traumatic	11	11
P15	29	M	4015	3920	15	C6	C	Traumatic	18	14
P16	31	M	530	495	13	C6	A	Traumatic	11	11
P17	29	F	750	690	10	C6	C	Traumatic	5	6
P18	52	F	3650	3550	10	C6	A	Traumatic	9	10
P19	61	F	9000	8930	14	C6	A	Traumatic	15	14
P20	40	M	400	310	10	C6	A	Traumatic	0	3
P21	41	M	3900	3825	15	C6	A	Traumatic	18	16
P22	36	F	4000	3940	14	C6	A	Traumatic	13	14
P23	22	F	3900	3830	14	C6	A	Traumatic	-	-
P24	65	M	230	190	10	C7	C	Traumatic	5	6
P25	22	M	290	230	10	C7	D	Traumatic	20	19
P26	47	M	1095	995	10	C7	D	Neoplastic	5	6
P27	62	F	300	260	10	T2	A	Myelitis	12	9
P28	34	M	240	215	13	T3	A	Traumatic	17	15
P29	42	M	1170	1140	11	T3	A	Traumatic	18	15
P30	47	M	2400	2330	11	T4	A	Traumatic	19	16
P31	56	M	2100	2020	13	T4	A	Traumatic	19	16
P32	53	M	10220	10160	14	T4	A	Traumatic	14	9
P33	72	F	188	160	11	T5	C	Traumatic	12	9
P34	44	M	6040	5985	15	T5	A	Traumatic	20	19
P35	63	M	4000	3880	10	T5	A	Neoplastic	20	15
P36	39	F	3850	3710	14	T5	A	Traumatic	20	19
P37	35	M	6770	6700	16	T7	A	Traumatic	20	19
P38	31	M	450	380	12	T7	A	Traumatic	20	17
P39	42	M	4945	4885	16	T8	A	Traumatic	20	19
P40	39	M	5840	5760	15	T8	A	Traumatic	19	16
P41	42	M	1170	1070	14	T9	A	Neoplastic	20	18
P42	42	M	4380	4320	15	T9	A	Traumatic	20	19
P43	19	M	789	750	14	T10	A	Traumatic	20	19
P44	42	M	960	925	15	T10	A	Traumatic	20	19
P45	35	M	590	520	11	T10	A	Traumatic	20	19
P46	27	M	1200	1170	14	T10	A	Traumatic	20	16
P47	40	M	4015	3865	15	T11	A	Traumatic	20	19
P48	47	M	2190	2140	15	T11	A	Traumatic	20	15
P49	39	M	3256	3195	14	T11	A	Traumatic	20	19
P50	49	F	450	410	11	T12	A	Traumatic	18	15
P51	38	M	2057	2000	15	T12	A	Traumatic	20	19
P52	29	M	2190	2160	16	L1	A	Traumatic	20	21
P53	56	M	600	540	10	L1	C	Traumatic	17	13
P54	34	M	1100	1055	12	L1	D	Neoplastic	20	21
P55	68	M	4745	4685	10	L1	A	Traumatic	17	17
Range	19 - 72	12 F - 43 M	188 - 10220	160 - 10160	10 - 16	C3 - L1	A - D	-	0 - 20	3 - 21
Mean	39.92		2768.76	2701.72	12.67				14.84	13.88
SD	12.65		2540.82	2533.73	2.15				6.42	5.17

Supporting Table 1. Clinical and demographic characteristics of patients with SCI.

The neurological levels of the lesion and injury, as determined by the American Impairment Scale (AIS), are indicated. Spinal Cord Independence Measure (SCIM) scores were not available for patient nos. 12 and 25.

Statement	Scores		Communalities		
	Mean	Standard deviation	1° component	2° component	3° component
Diet	3.18	2.15	0.05	0.54	0.57
Maintenance	4.45	2	0.001	0.66	0.67
Defense	4.03	2.37	0.002	0.61	0.66
Tool	3.2	2.38	0.32	0.36	0.66
Affect	2.9	2	0.27	0.37	0.63
Entire body	2.6	2.34	0.02	0.03	0.82
Lower limbs	3.38	2.58	0.58	0.66	0.68
Substitution	3.74	2.77	0.53	0.53	0.54
Action	5.12	2.24	0.53	0.68	0.68

Supporting Table 2:Scores and communalities on questionnaire statements.

Mean, standard deviations, and communalities for the three components in each of the statements.
